# Reformulating ice cream to improve postprandial glucose response: an opportunity for industry to create shared value

**DOI:** 10.3389/fnut.2024.1349392

**Published:** 2024-07-16

**Authors:** Ebaa Al-Ozairi, Yousef Mandani, Ghanima Alfaleh, Jincy Raj, Shadan Alshammari, Carel W. Le Roux

**Affiliations:** ^1^Clinical Research and Clinical Trials Unit, Dasman Diabetes Institute, Kuwait, Kuwait; ^2^Ministry of Health, Kuwait, Kuwait; ^3^Department of Food Science and Nutrition, College of Life Sciences, Kuwait University, Kuwait, Kuwait; ^4^Diabetes Complications Research Centre, University College Dublin, Dublin, Ireland

**Keywords:** creating shared value, metabolic matrix, no-added sugar ice cream, postprandial glucose responses, type 2 diabetes

## Abstract

Ultra-processed foods are associated with metabolic dysfunction and driving chronic diseases. The Metabolic Matrix is a tool used to reformulate products to promote positive metabolic outcomes. The Kuwait Danish Dairy Company (KDD) has used this tool to develop a no-added-sugar products. This clinical trial tested the glycaemic response of a no-added-sugar ice cream in individuals with type 2 diabetes. The hypothesis was that the no-added-sugar ice cream would have a substantially better postprandial glycemic response than conventional ice cream in patients with type 2 diabetes. In this randomized cross over designed study, postprandial glycemic response was measured after 300 grams of no-added-sugar ice cream or normal ice cream was consumed. Despite similar composition and palatability, the postprandial responses were better with the no-added sugar ice cream, albeit that the natural sugar in the product still resulted in a marked postprandial glycaemic response. This finding emphasizes the necessity of clearly communicating to both patients and healthcare professionals that “no-added-sugar” does not equate to “zero total sugar.” The path to improved metabolic health involves not only product improvement but also transparent messaging to enable informed dietary choices. Reformulation resulting in palatable no-added sugar products provides an opportunity for companies to Create Shared Value by addressing the important social problems such as obesity and type 2 diabetes, by creating scalable solutions, that are profitable.

**Clinical trial registration:**ClinicalTrials.gov, identifiers NCT06135935.

## 1 Introduction

Ultra-processed foods negatively impact human and environmental health and are associated with various adverse health outcomes, communicable and non-communicable diseases such as type 2 diabetes, obesity, and cardiovascular disease (1). Type 2 diabetes as a consequence of the disease of obesity is a global challenge (2), while reducing lifespan and quality of life. Moreover type 2 diabetes consumes more than 10% of the health budgets of most developed countries (3).

The Metabolic Matrix is a science-based tool used for reformulating products with the aim of promoting positive metabolic outcomes (4). By using this tool, the food and beverage industry can make informed decisions to create products that are palatable, desirable, healthier and more beneficial for metabolic health. The food and beverage industry needs to use food technology to address important social problems such as metabolic diseases (5), by using food reformulation, portfolio evolution, preserving the food matrix, encouraging transparency, and improving food environments (6).

Porter et al. introduced the concept of “Creating Shared Value” in a Harvard Business Review article (7). The central idea behind Creating Shared Value is that businesses can support a triple bottom line, creating economic value while addressing social and environmental challenges. Traditionally, the role of a corporation has been seen as creating value primarily for its shareholders. However, the approach of Creating Shared Value argues that companies can ensure long-term financial success by identifying and addressing social and environmental needs through their core business operations and products. In doing so, they can simultaneously create value for themselves *and* society at large, achieving a competitive advantage that goes beyond corporate responsibility and sustainability. Creating Shared Value is different from Corporate Social Responsibility. While Corporate Social Responsibility focuses on doing good as a separate or supplementary aspect of business, Creating Shared Value seeks to intertwine business success with social progress, creating a mutually beneficial relationship.

The Creating Shared Value framework emphasizes three main ways that companies can Create Shared Value. Firstly, by redefining the purpose of the company. Instead of just focusing on profit maximization, businesses can adopt a broader purpose that includes addressing social and environmental issues. By aligning their core business strategies with societal needs, they can Create Shared Value for all stakeholders. Secondly, by expanding the value chain, companies can look beyond their immediate operations and influence the value chain to create social benefits. This involves working with suppliers, distributors, and other partners to improve practices and outcomes throughout the supply chain. Thirdly, by enhancing the local context, businesses can make a positive impact by investing in the communities where they operate. This can include creating scalable and profitable solutions for education, healthcare, infrastructure, and other initiatives that improve the well-being of local residents.

The concept of Creating Shared Value has gained traction over the years, but it is different from corporate social responsibility and sustainability strategies. By integrating societal needs into their business models, companies can create a more sustainable and mutually beneficial relationship between business and society, in contrast to corporate social responsibility where the company does not immediately benefit financially.

More specifically, Creating Shared Value thinking has been adopted in the food and beverage industry to enhance competitiveness while advancing social and economic conditions in the communities where the company sells and operates (8). A key concern with current food systems is that the true costs of unhealthy foods are externalized, not reflected in market prices, and paid for by society at large. Current externalities have been estimated to be nearly double (19.8 trillion USD) the current total global food consumption (9 trillion USD) (9). The sum of externalities includes seven trillion USD in environmental costs, 11 trillion USD in costs to human life, and one trillion USD in economic costs. In other words, for every dollar in value the food and beverage industry produces, two dollars in harm is incurred (9).

The Kuwait Danish Dairy Company (KDD) (10) has developed a line of no-added-sugar products using a methodology for reformulating products to maximize their positive health impacts using the Metabolic Matrix tools (4). A range of new product developments include ice creams that have no added sugar, resulting in a significant reduction in net carbohydrates. The glycaemic impact, especially in patients with type 2 diabetes, was however unknown. It is important to note that ice cream contains milk which has carbohydrates coming from lactose, which is a sugar. The total carbohydrate in milk is therefore naturally occurring sugars from lactose. Milk is not naturally sweet tasting unless sugar from products such as cane or other sugars, high fructose corn syrup, or other sweet sources are added, which was the case with the traditional KDD ice cream. The no added sugar KDD ice cream thus contained artificial sweeteners.

Two out of 39 criteria in the Metabolic Matrix feature sugar reduction, calling for no more than 2 grams of fructose and 4 grams of glucose per serving in newly developed products. Precise and evidence-based criteria indicate that the sugar reduction ambitions depart from conventional efforts in the food and beverage industry that typically only report sugar reduction at the portfolio level and lack quantified targets at the product or per-serving scale. Thus the sugar reduction strategies include not adding sugar, using safe non-caloric sweeteners and sweetener modifiers (4).

The hypothesis was that the no-added sugar ice cream would have a substantially better postprandial glycaemic response than conventional products in patients with type 2 diabetes. The aim of this double-blind clinical trial was to measure the impact on postprandial glycaemia response of consuming 300 g of no added sugar vs. conventional ice cream, since, from an independent nutrition perspective, the ice creams appear similar in composition.

## 2 Methods

The primary endpoint for this randomized cross over designed clinical trial was the area under the curve for the plasma glucose response over 120 min after consumption of two ice creams which varied in added sugar. The secondary outcomes included: 1. plasma insulin responses over 120 min after consumption of the ice creams, 2. continuous glucose monitoring over 24 h after consumption of the ice creams, and 3. palatability scores of patients on visual analogue scales.

The population included patients with type 2 diabetes, over the age of 18 years, with a HbA1c > 6.5% (48 mmol/L). The usual medications of the patients were unchanged on both occasions. Patients were excluded if they were lactose intolerant or did not like chocolate ice cream. The Dasman Diabetes Institute Ethics committee provided approval (RA HM 2023–002) and all patients provided written informed consent. The study was conducted according to the Principals of Helsinki and registered at ClinicalTrials.gov (Identifiers: NCT06135935).

The power calculation suggested that twelve participants who are randomized in a cross-over design will provide ≥90% power at the 1.7% significance level for the detection of a two-fold difference in the achievement of the primary outcome between groups. The ɑ level was set at 1.7%. A *p*-value of <0.05 was considered as significant.

Randomization and concealed assignment were implemented, and both the researchers involved with the study visits and the subjects were blinded. During the visits, the patient arrived at 09.00 after an overnight fast of at least 6 h. Patients were canulated and 10 mL of blood was obtained immediately before the meal was consumed and then 15, 30, 60, 90, and 120 min after the meal was started. Patients consumed 300 g of ice cream according to their randomized assignment. The study employed a crossover design, with participants having a 7-day washout period before consuming the two ice creams. Visual analogue scales were also completed before the start of the meal and at 15, 30, 60, 90, and 120 min thereafter. After 120 min, patients returned home with a continuous glucose monitoring device in place. The routine laboratory measured plasma glucose and insulin. Supplementary Table S1 provides the nutritional facts for no-sugar-added ice cream and regular ice cream.

## 3 Results

The demographic and clinical characteristics of the participants are presented in Table 1. All 12 patients completed both visits. Ten patients were able to consume all 300 g of the ice cream on both occasions. Two patients were not able to consume all the ice cream on the first occasion. The weight of the ice cream they consumed was measured (95 g and 150 g), and these participants were asked to eat the same amount of ice cream on the second visit. The analysis thus remained paired because all participants consumed the same amount of ice cream on both occasions.

**Table 1:** Demographic and clinical characteristics of the participants.

	Mean (SD) or *n* (%)
Age (years)	58.0 (12.1)
Male	8 (66.7%)
Diabetes duration (years)	9.9 (7.9)
BMI (kg/m2)	31.0 (5.7)
Waist Circumference (cm)	106.1 (13.4)
Systolic BP (mmHg)	127.8 (10.9)
Diastolic BP (mmHg)	78.0 (7.6)
Fasting blood glucose (mmol/L)	7.4 (1.7)
HbA1c (%)	7.3 (0.6)
eGFR (mL/min)	86.2 (27.9)
Urine ACR (mg/g)	45.9 (62.5)

**Figure 1:**
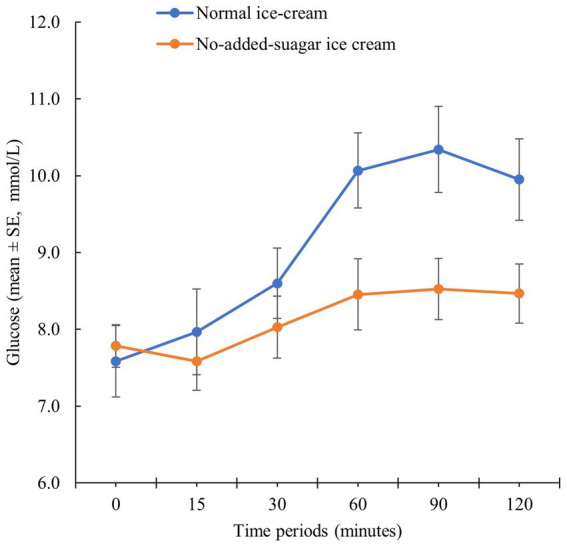
Postprandial glucose response.

The primary outcome was the area under the curve for glucose over 120 min and the no-added-sugar ice cream had a better glycaemic profile (*p* = 0.003, Figure 1). The blood glucose at 120 min was lower during the no-added-sugar ice cream meal (8.5 ± 1.3 mmol/L vs. 10.0 ± 1.8 mmol/L, *p* = 0.016). The peak blood glucose during any time over the 120 postprandial minutes was also lower during the no-added-sugar ice cream meal (8.7 ± 1.4 mmol/L vs. 10.5 ± 1.8 mmol/L, *p* = 0.002 respectively).

The secondary outcome was the area under the curve for insulin over 120 min, and the no-added-sugar ice cream was better (2197.1 ± 867.9 min.mmol/L) compared with the regular ice cream (3336.7 ± 1232.0, *p* = 0.002). The plasma insulin at 120 min was 19.3 ± 9.0 IU/L for no-added-sugar ice cream vs. 34.5 ± 18.0 IU/L for normal ice cream (*p* = 0.004). The peak plasma insulin during any time over the 120 postprandial minutes was 21.8 ± 9.4 for no-added-sugar ice cream meal and 37.3 ± 17.8 IU/L for normal ice cream (*p* = 0.002). The continuous glucose monitoring showed mean glucose levels over 120 min of 7.8 ± 1.4 mmol/L for no added-sugar ice cream and 9.0 ± 1.7 mmol/L for normal ice cream (*p* = 0.006). No adverse events were reported during the study, and no hypoglycaemic episodes were recorded during the continuous glucose monitoring. There was no difference in the time in range measured over 7 consecutive days before and after the consumption of normal ice-cream and no-added sugar ice cream (*p* = 0.24).

## 4 Discussion

Creating Shared Value addresses critical social needs, as companies strive to create scalable solutions. This approach emphasizes that businesses can achieve economic success while contributing positively to society. By focusing on scalable solutions, companies can expand their efforts to reach a broader population and have a more significant impact on addressing social challenges. Scalability allows these solutions to be replicated and applied in different contexts, making them more sustainable in the long term.

Increasing concerns about ultra-processed foods and their role in non-communicable diseases (11) and communicable diseases (12) highlight the urgent need for the food and beverage industry to develop product innovations and reformulations that increase the availability of healthy foods and support more sustainable food systems (13). However, independent nutrition research is needed to validate these efforts, requiring collaboration between academia and the food and beverage industry (14).

The emphasis on profitability is essential because it aligns with the company’s financial interests and without it social needs will not be addressed. Profitable solutions ensure the initiatives can be self-sustaining and do not rely solely on philanthropic funding. A business model incorporating social impact as a core aspect creates a self-reinforcing mechanism where social progress drives business success and vice versa. This approach challenges the traditional view that social and economic goals conflict. Instead, it suggests that businesses can find opportunities to address societal challenges, leading to a win-win situation for the company and the communities they serve.

Some companies in the food and beverage sector have promoted the idea that personal responsibility and excess calories are the sole cause of diet-related chronic diseases. This approach has fostered epidemics of metabolic disorders, impacting quality of life, medical resources, and healthcare budgets around the world. These impacts are very significant in regions such as the Middle East and North Africa (15). By taking a different approach and focusing on metabolic impacts, not merely the nutrition facts on product labels, more benefit may be achieved. The Metabolic Matrix model can be used by any food and beverage company to develop healthier products (4).

Adopting a Shared Value strategy requires a shift in mindset and corporate culture. It involves a deep understanding of social issues, collaboration with stakeholders, and innovative thinking to develop sustainable and profitable solutions that create lasting value for all involved parties. Historically, emphasis was placed on consumer behavior and lifestyle choices as leading factors in driving change in the marketplace. This was because diet interventions that seek to alter the ‘external’ setting or choice environment are more effective than those that target ‘internal’ motivations (16).

Using a very conventional scientific approach to show the effect of a palatable no-added-sugar ice cream can have on postprandial glucose curves provides endorsement of a potential scalable solution. The small number of patients required to show the effect is a testament to the consistency of the effect and the effect size. The postprandial curves of the no-added-sugar ice cream show that there is still a significant amount of natural sugar in the product. It will be essential to emphasize to patients and healthcare professionals that “no-added-sugar” does not mean “no sugar.”

The limitations of this study include that the small number of participants did not adequately allow palatability to be assessed. Although no differences in palatability between the no-added-sugar ice cream and the usual ice cream were detected, a much larger cohort would be needed to adequately determine consumer satisfaction and all the aspects of palatability.

In conclusion, the no-added-sugar ice cream resulted in lower postprandial glucose curves. The palatability of the product does make it a candidate for Creating Shared Value because it can help address critical social problems, such as type 2 diabetes and obesity, by creating scalable solutions that may be profitable.

## Data availability statement

The raw data supporting the conclusions of this article will be made available by the authors, without undue reservation.

## Ethics statement

The studies involving humans were approved by the Dasman Diabetes Institute Ethics committee, Kuwait. The studies were conducted in accordance with the local legislation and institutional requirements. The participants provided their written informed consent to participate in this study.

## Author contributions

EA-O: Conceptualization, Data curation, Formal analysis, Funding acquisition, Investigation, Methodology, Project administration, Resources, Supervision, Validation, Visualization, Writing – original draft, Writing – review & editing. YM: Data curation, Methodology, Writing – review & editing. GA: Conceptualization, Data curation, Investigation, Methodology, Supervision, Writing – original draft, Writing – review & editing. JR: Data curation, Methodology, Writing – review & editing. SA: Data curation, Methodology, Writing – review & editing. CWLR: Conceptualization, Formal analysis, Investigation, Methodology, Software, Supervision, Validation, Visualization, Writing – original draft, Writing – review & editing.
