# Supplementary material for: Reformulating ice cream to improve postprandial glucose response: an opportunity for industry to create shared value
Source: Front Nutr. 2024 Jul 16;11:1349392. doi: 10.3389/fnut.2024.1349392 (PMC11288177; doi:10.3389/fnut.2024.1349392)
Supplement: Supplementary file 1 [file Table_1.DOCX]

Supplementary Table 1: Nutritional facts of no-sugar-added ice cream and regular ice cream per 100g serving size.

| Nutritional facts per 100g serving size | No-sugar-added ice cream | Regular ice cream |
| --- | --- | --- |
| Total fat (g) | 10 | 10 |
| Saturated fat (g) | 7 | 9 |
| Cholesterol (mg) | 20 | 5 |
| Sodium (mg) | 45 | 40 |
| Total carbohydrate (g) | 22 | 24 |
| Total sugar (g) | 6 | 21 |
| Sugar alcohol (g) | 7 | - |
| Added sugar (g) | - | 16 |
| Protein (g) | 4 | 3 |
| Calcium (mg) | 100 | 90 |
| Iron (mg) | 0.7 | 0.7 |
| Potassium (mg) | 310 | 290 |
